# BCL-2 and MYC gain/amplification is correlated with central nervous system involvement in diffuse large B cell lymphoma at leukemic phase

**DOI:** 10.1186/s12881-017-0381-z

**Published:** 2017-02-16

**Authors:** Dehui Zou, Shuhua Yi, Rui Cui, Wei Liu, Chengwen Li, Shizhen Zhong, Zhen Yu, Zengjun Li, Rui Lv, Kun Ru, Huijun Wang, Gang An, Yan Xu, Lugui Qiu

**Affiliations:** 1 State Key Laboratory of Experimental Hematology, Institute of Hematology and Blood Disease Hospital, Chinese Academy of Medical Sciences and Peking Union Medical College, No.288, Nanjing road, Heping district, Tianjin 300020 China; 2 Department of Hematology, Tianjin First Center Hospital, Tianjin, China

**Keywords:** Diffuse large B cell lymphoma, Leukemic phase, Centre nervous system involvement, BCL2, MYC

## Abstract

**Background:**

Diffuse large B-cell lymphoma (DLBCL) of leukemic phase is a rare clinical manifestation, but is highly prevalent with central nervous system involvement (CNSI). Little is known about this rare clinical observation.

**Methods:**

We reviewed the clinical characteristics of 40 DLBCL patients with leukemic phase identified by flow cytometry and analyzed *BCL2* and *MYC* aberrations by fluorescence in situ hybridization.

**Results:**

The median age of these 40 patients was 46 years (range, 15–75) with 19 men patients. All patients had bone marrow involvement, and fourteen (35.0%) had CNSI. There were respectively 14 patients (35.0%) had the BCL2 or MYC gain/amplification and nine of them (22.5%) simultaneously had both aberrations. Compared to those without CNSI, CNSI was found more commonly in male patients (71.4 vs. 34.6%, *p* = 0.046), in those with IPI scores of 4–5 (57.1% vs. 11.5%, *p* = 0.001), and in those with elevated serum LDH (100 vs. 61.5%, *p* = 0.007) and both MYC and BCL2 rearrangement (88.9 vs. 19.4%; *p* = 0.000). BCL2 and MYC rearrangements were the sole independent factor correlated with CNSI.

**Conclusion:**

It is possible that both BCL2 and MYC gene aberrations may contribute to the high incidence of CNSI observed in leukemic phase of patients with DLBCL.

## Background

Diffuse large B-cell lymphoma (DLBCL) is the most common lymphoid neoplasm, which account for 30%-40% of non-Hodgkin lymphoma (NHL) [1] in the Western countries and more than 50% of NHL in China. DLBCL is a heterogeneous group of disorders with variable histological and clinical behavior. Up to 40% of patients may have extranodal involvement at diagnosis. The common extranodal sites include the gastrointestinal tract, bone, bone marrow, testis, salivary gland, adrenal gland, liver, kidney, and central nervous system (CNS) [1]. The extent of extranodal involvement can impact the overall prognosis of each patient [2]. For example, patients with CNS involvement, which is observed in up to 2%-8% of patients, have poorer outcomes [2–5]. Tumor cells circulated in the peripheral blood (PB) occur rarely in patients with DLBCL. However, approximately 16% of patients at leukemic phase have CNS involvement, which is associated with increased mortality [6]. It remains unknown why patients with leukemic phase have a higher incidence of CNS involvement. As the double hit with MYC and BCL2 gene rearrangements contribute to the high aggressive behavior of DLBCL [7], we analyzed the cytogenetic aberrations of patients with DLBCL at leukemic phase. We observed that both BCL-2 and MYC genes gain/amplification were strong independent indicators of CNS involvement in DLBCL patients at leukemic phase.

## Methods

### Patients

A flow cytometry database was searched to identify patients with NHL and with circulating lymphoma cells between August 2001 and May 2012 at Institute of Hematology and Blood Disease Hospital, Chinese Academy of Medical Sciences and Peking Union Medical College (CAMS & PUMC). The target antigens for flow cytometry included CD19, CD20, CD22, CD10, CD23, FMC7, kappa, lambda, CD3, CD5, CD45, CD11c, CD103 and sIgM. All antibodies were obtained from BD Biosciences.

Finally, forty patients with de novo DLBCL and with complete medical records were enrolled in this study. Patients with secondary DLBCL were excluded by the clinical course and medical history. Basic demographics (age, gender), performance status, Ann Arbor stage, presence of extra nodal sites, chromosome karyotype, bone marrow biology and peripheral blood morphology, cerebrospinal fluid (CSF) involvement, hematological parameters, lactate dehydrogenase (LDH) levels, and treatment outcomes were assessed. Histologic specimens were reviewed by hemato-pathologists, including two of the authors of this study, based on WHO classification [1]. All bone marrow tissues evaluated in this study were performed at the time of initial staging. All patients enrolled gave informed consent in accordance with the Declaration of Helsinki. The study was approved by the Ethics Committee of CAMS & PUMC.

CNS involvement was defined based on the combination of clinical CNS manifestations, radiological findings, and/or examination of the cerebrospinal fluid (CSF). Cytology study of CSF and CNS imaging by brain magnetic resonance imaging (MRI)/computerized tomography (CT) were performed for patients with a clinical suspicion of CNS involvement. The patterns of CNS involvement were as follows: 3 patients identified as parenchymal involvement, 9 patients with leptomeningeal involvement and 2 patients with combined involvement. Five patients were presented with CNS involvement at diagnosis and others at progression or disease recurrence.

### Fluorescence In Situ Hybridization (FISH)

Interphase FISH analysis was performed on bone marrow samples at diagnosis. The DNA probes used were LSI BCL2 and MYC Dual Color, Break Apart Rearrangement Probe, LSI IGH/BCL2 Dual Color, Dual Fusion Translocation Probe and LSI IGH/MYC/CEP 8 Tri-Color Dual Fusion Probes (purchased from Vysis, USA). Sample preparations and hybridizations were conducted following manufacturer’s recommendations. Methods for FISH analysis are described elsewhere [8, 9]. At least 200 cells with well delineated signals were evaluated. The cut-off for positive values (mean of normal control + 3SD), determined from samples of ten cytogenetically normal persons, were 4.5% for LSI BCL2 and MYC Dual Color, Break Apart Rearrangement Probe and 3.2% for LSI IGH/BCL2 Dual Color, Dual Fusion Translocation Probe and LSI IGH/MYC/CEP 8 Tri-Color Dual Fusion Probes. Gains were defined as three copies of the gene studied, whereas at least four copies were considered as amplifications [10].

### Survival and statistical analysis

Overall survival (OS) was measured as the interval between the date of treatment and the date of death or last follow-up. Progression-free survival (PFS) was measured as the interval between the date of treatment and the date of death from any cause or disease progression. Fisher’s exact test or chi-square test was used to determine statistically significant differences between the clinical characteristics of the two groups. Survival curves were constructed by the Kaplan-Meier method, and prognostic features were evaluated on univariate analysis (log-rank test). The effects of potential prognostic variables on survival were assessed according to the Cox regression method. P values <0.05 were considered statistically significant. All calculations were performed using the SPSS statistical software package (Version 13.0).

## Results

### Clinical characteristics

The clinical characteristics of the 40 patients are presented in Table 1. In this study, there were a large number of younger patients, with a median age of 46 years (range 15–75). The median white blood cell (WBC) was 12.32 × 10^9^/L, while the median percentage of circulating lymphomatous cells was 35.74% (range, 1–90) as determined by flow cytometry. Twenty-seven patients (67.5%) had anemia and half of these patients had reduced platelet at diagnosis. All of the 40 patients had bone marrow involvement, and 14 patients had CNS involvement. Other extranode sites involvement included lung, kidney, pancreas, adrenal gland, liver, testis, and bowel.

**Table 1:** the comparison of clinical characteristics between patients with or without CNS involvement

Characteristics	With CNS involvement	Without CNS involvement	*P* value
	N=14	N=26	
Age, median (range, year)	44.0 (23.0-62.0)	51.0(15-75)	.392
Gender, men, n(%)	9 (34.6%)	10(71.4%)	.046
B symptoms, n (%)	10 (71.4%)	15(57.7%)	.502
Elevated LDH, n (%)	14 (100%)	16(61.5%)	.007
Median WBC×109/l (range)	17.2 (1.2-66.57)	9.58(1.38-40.06)	.729
Median Hb, g/l (range)	95.0 (61.0-145.0)	100.5(45.0-141.0)	.777
Median PLT, ×109/l (range)	73.5 (20.0-355.0)	113.0(3.0-509.0)	.843
International Prognostic			.002
Index, n (%)	0	9 (34.6%)	
2	6 (42.9%)	14(53.8%)	
3	8 (57.1%)	3(11.5%)	
4-5			.896
Treatment, n (%)	4 (28.6%)	10(38.5%)	
CHOP/CHOP-like	1 (7.1%)	3(11.5%)	
R- CHOP/CHOP-like	4 (28.6%)	5(19.2%)	
Intensive regimes	5 (35.7%)	8(30.8%)	

As there was a high percentage of CNS involvement in these patients at leukemic phase, we compared the clinical characteristics between patients with or without CNS involvement (Table 1). There was a significant male preference, higher elevated LDH percentage and high risk IPI group in patients with CNS involvement. The median age of the patients with CNS involvement was also younger than those without (44 vs. 51 years, *p* = 0.392).

### Cytogenetic aberrations

**Fig. 1:**
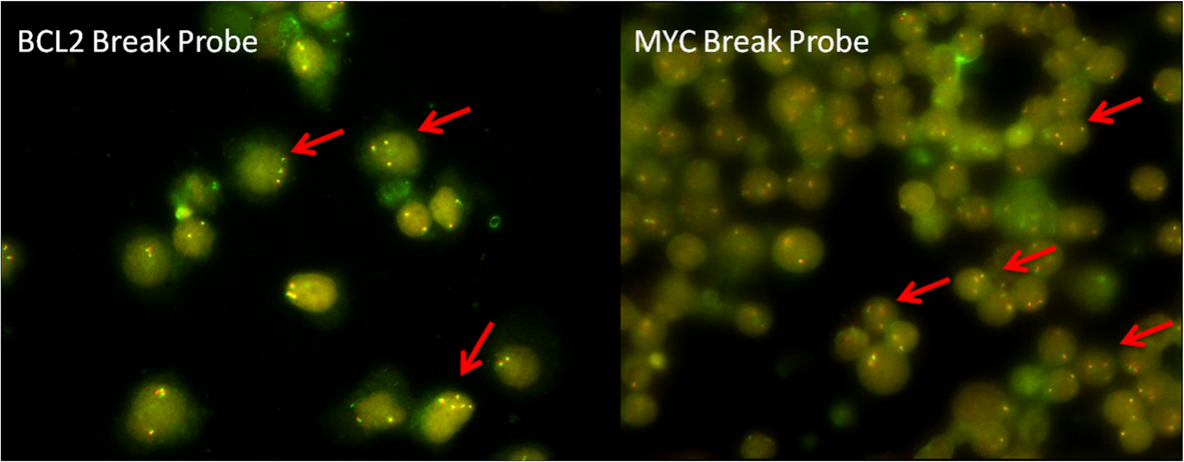
Genetic aberrations of BCL2 and MYC gene as detected by FISH. The red arrow indicated gene gain or amplification (three or more fusion signals)

Using the bone marrow cells, we detected *BCL2* and *MYC* gene arrangements by FISH. There was no t (14;18) (q32;q21) abnormality detected by BCL2/IGH Dual Fusion Translocation Probe. However, 14 patients had three or more fusion signal of BCL2 (Fig. 1), which meant that 35% patients had *BCL2* gene gain/amplification. Additionally, gain/amplification of MYC was presented in 14 patients (Fig. 1), with one having concomitant MYC/IGH translocation. Nine patients exhibited both *MYC* and *BCL2* gene gains/amplifications. As shown in Fig. 1, gain and amplification always concurred in one patient. Some patients display predominant amplifications with minor gains and others reversely. So we did not discriminate gain and amplification in an individual here and put gain and amplification together to analyze and used “abnormality” instead of “gain” or “amplification” in this manuscript.

Eight of the fourteen patients (57.1%) with BCL2 abnormality had CNS involvement, while 6 of 26 patients (23.1%) that lacked the BCL2 abnormality had CNS involvement (*p* = 0.043). Eleven of the 14 patients (78.6%) with MYC abnormality had CNS involvement, which was significant higher than three of the 26 patients (11.5%) without MYC abnormality (*p* < 0.001). Among the 9 patients with both MYC and BCL2 abnormality, eight patients had CNS involvement, while only 6 of the other 31 patients with BCL2 or MYC or no abnormality had CNS involvement (88.9 vs. 19.4%; *p* < 0.001).

Other clinical characteristics that were associated with CNS involvement (as shown above) included gender, elevated LDH, and high risk IPI group. We performed a multivariate analysis to determine the relationship between the clinical characteristics and CNS involvement, by integrating gender, elevated LDH, high risk IPI, and concomitant BCL2 and MYC abnormalities. We demonstrated that concomitant BCL2 and MYC abnormalities were the only independent factor that correlated with CNS involvement (relative risk 15.3, 95% confidence interval, 1.4-171.1, *p* = 0.027). Therefore, there was a strong association between DLBCL patients with concomitant of BCL2 and MYC abnormalities and CNS involvement.

### Treatment and survival

All of the 40 patients included in this study had received at least 2 cycles of chemotherapy, with median 4 cycles (range 2–10). As shown in Table 1, fourteen patients had received CHOP or CHOP-like regimens, while nine patients had received HyperCVAD/MA alternating chemotherapy or CHOPE/ EPOCH regimens chemotherapy. Seventeen patients had received rituximab combination chemotherapy, including 4 patients with R-CHOP/CHOP-like regimens and other with R-intensive regimens. Three patients received R-HyperCVAD/MA regimens as inductive chemotherapy and then took autologous stem cell transplantation (ASCT) as consolidation treatment. Intrathecal chemotherapy was done in patients with CNS involvement. Eleven patients (27.5%) had reached partial remission (PR), and fifteen patients (37.5%) with complete remission or uncertain complete remission (CR/CRu). Therefore, the overall response rate (ORR) was 62.5%. The ORR and CR/CRu for the 17 patients treated with rituximab was 76.4% and 52.9% respectively.

With a median follow-up of 18 months (range 2–93 months), 32 patients had died and the median follow-up for the surviving patients was 34.5 months (range 22–69 months). The median PFS and OS was 11.0 (95% CI, 6.0-16.0) and 18.0 (95% CI, 8.7-27.3) months, respectively. The median PFS and OS of the patients with CNS involvement were 5.0 (95%CI, 4.1-5.9) and 8.0 (0.7-15.3) months, respectively, which were significantly shorter than those without CNS involvement (median PFS, 25.0 [range, 11.1-38.9 months], *p* < 0.001; OS, 29.0 [range, 24.5-33.5 months], *p* = 0.01). DLBCL patients with concomitant *BCL2* and *MYC* abnormalities also had a shorter median PFS (5.0 vs.22.5, *p* = .002) and OS (9.0 vs.27.5, *p* = .001) than those without the genetic abnormality. Two of the patients receiving ASCT had disease progression after three months of ASCT, with one patient surviving over five years.

## Discussion

Peripheral blood involvement in patients with DLBCL is rare. Currently, there are only three large studies have reported the frequency of PB involvement in DLBCL patients. In a Japanese study, 1.2% of patients with DLBCL demonstrated PB involvement [2], while two other studies from Western countries showed that 4%-5.3% of patients with DLBCL exhibited PB involvement [11, 12]. However, morphologic examination of peripheral blood smears revealed that approximately one third of DLB CL patients with BM involvement also had malignant cell in PB. These studies did not assess the association between clinical and cytogenetic characteristics and PB involvement in DLBCL patients. To our knowledge, this study is the largest to report the clinical characteristics and outcome of DLBCL patients at leukemic phase [6, 13].

Similar clinical characteristics of leukemic DLBCL were identified in the study by Murungampurath-John [6], including the median age, gender, B symptom, bone marrow involvement, median WBC and HB, and the distribution of IPI index. However, in our study, the number of extranodal site involvement was lower than that of a study by Murangampurath-John (50 vs. 100%) [6]. CNS involvement was reported to be 22% in the study by Muringampurath-John [13], which is lower than that reported in our study (35%). However, the reported number of CNS involvement in our study and the study by Muringampurath-John was higher than that reported in other DLBCL patient populations. When comparing the clinical characteristics between patients with or without CNS involvement, age was associated with CNS involvement. Additionally, when compared to the study by Muringampurath-John, the median WBC and PLT, and the percentage of circulating lymphomatous cells in this study were comparable, except for a higher percentage of patients with high IPI scores (≥4) in that study (41 vs. 27.5%) [6].

Cytogenetic aberrations are biologic and diagnostic hallmarks of mature B-cell lymphomas. The t (14;18) (q32;q21) translocation is the most common translocation observed in follicular lymphomas [1], which could potentially transform to DLBCL, [14]. In this study, 35% DLBCL patients had *BCL2* gene gain/amplification and none were reported having the t (14;18) translocation, which was consistent with no patients having FL history. Recently, it was demonstrated that a double-hit lymphoma caused by multiple genetic aberrations, such as the MYC/8q24 locus and BCL2/18q21.3 locus can give rise to a unique subset of lymphomas. Translocation or amplification of the *BCL2* gene occurred in 20-30% of cases of reported lymphomas [15]. *MYC* rearrangement have been reported in up to 10% of an unselected series of cases and is associated with a complex pattern of genetic alterations [1]. Most of the *MYC* translocations occur with IG genes [16]. However, these cytogenetic aberrations have not been specially detected in DLBCL patients at leukemic phase. Moreover, gene rearrangement was the main aberration of BCL2/MYC in DLBCL other than gene gain/amplification. Some studies have also reported that patients with 18q21.3/BCL2 and 8q24/MYC genetic rearrangement are at higher risk of having CNS involvement [16–18]. The incidence of CNS involvement ranged from 9% to 50% in double-hit (DH) lymphoma [7, 19, 20]. In this study, the incidence of CNS involvement increased to 88.9% in DLBCL patients at leukemic phase with concomitant *BCL2* and *MYC* gain/amplification. However, for patients with primary lymphoma of the CNS (PCNSL), it was reported that up to 8% had *MYC* rearrangement and none with *BCL2* rearrangements [21]. Therefore, the underlying mechanism of CNS involvement of leukemic DLBCL may differ from PCNSL.

PB or CNS involvement or concomitant genetic abnormalities of *MYC* and *BCL2* have been reported to be associated with poor survival in DLBCL [2, 16, 18]. Patients with leukemic phase have lower CR rate (44%) even after rituximab combination chemotherapy, indicating drug-resistance for this population [13]. In this study, the CR/CRu rate for the patients treated with rituximab was 52.9%, comparable to previous report (54%) [6]. In this study, the median OS for all patients was 18 months, with CNS involvement and both BCL2/MYC dual abnormalities being predictors of poor clinical in DLBCL patients of leukemic phase. Three patients had received ASCT in this study. Two patients with CNS involvement reached CR/CRu after R-HyperCVAD/MA introductive chemotherapy but had disease progression even after ASCT. The other patient remains alive after ASTC, and did not have CNS involvement. This phenomenon indicates that new chemotherapy or targeted therapy is needed for these patients.

## Conclusions

Our study demonstrated that patients with DLBCL of leukemic phase had higher incidence of CNS involvement and concomitant BCL2 and MYC gene gains/amplifications. The concomitant of BCL2 and MYC gene gains/amplifications was the only independent factor that correlated with CNS involvement. Additionally, these patients exhibited a poorer treatment response and survival despite combination therapy with rituximab and ASCT.

## Abbreviations

ASCT: Autologous stem cell transplantation
CHOP: Cyclophosphamide, doxorubicin, vincristine, prednisone
CNS: Central nervous system
DLBCL: Diffuse large B-cell lymphoma
FISH: Fluorescence in situ hybridization
Hb: Hemoglobin concentration
IPI: International prognostic index
LDH: Lactate dehydrogenase
NHL: Non-Hodgkin lymphoma
OS: Overall survival
PB: Peripheral blood
PFS: Progression-free survival
PLT: Platelet number
R: Rituximab
WBC: White blood cell
